# Advances in Video Emotion Recognition: Challenges and Trends

**DOI:** 10.3390/s25123615

**Published:** 2025-06-09

**Authors:** Yun Yi, Yunkang Zhou, Tinghua Wang, Jin Zhou

**Affiliations:** 1 School of Mathematics and Computer Science, Gannan Normal University, Ganzhou 341000, China; 2 Key Laboratory of Data Science and Artificial Intelligence of Jiangxi Education Institutes, Gannan Normal University, Ganzhou 341000, China

**Keywords:** video emotion recognition, psychological models, datasets, algorithms, emotional representation

## Abstract

Video emotion recognition (VER), situated at the convergence of affective computing and computer vision, aims to predict the primary emotion evoked in most viewers through video content, with extensive applications in video recommendation, human–computer interaction, and intelligent education. This paper commences with an analysis of the psychological models that constitute the foundation of VER theory. The paper further elaborates on datasets and evaluation metrics commonly utilized in VER. Then, the paper reviews VER algorithms according to their categories, and compares and analyzes the experimental results of classic methods on four datasets. Based on a comprehensive analysis and investigations, the paper identifies the prevailing challenges currently faced in the VER field, including gaps between emotional representations and labels, large-scale and high-quality VER datasets, and the efficient integration of multiple modalities. Furthermore, this study proposes potential research directions to address these challenges, e.g., advanced neural network architectures, efficient multimodal fusion strategies, high-quality emotional representation, and robust active learning strategies.

## 1. Introduction

Emotion is a psychophysiological phenomenon [1,2,3]. Video emotion recognition (VER), also known as affective video content analysis, is an interdisciplinary domain where affective computing and computer vision intersect. The objective of VER is to predict the principal emotion of most audiences after they watch a video. VER has received extensive attention on account of its wide-ranging applications [4,5,6], including video recommendation, human–computer interaction, intelligent education, and advertising effectiveness evaluation. By analyzing audiovisual cues, VER systems infer the emotional responses of viewers, enabling more personalized and emotionally intelligent technologies.

The origins of VER are rooted in the pioneering studies of affective computing. Initially focused on recognizing emotions from static images or audio signals, research gradually extended to video data as computational power and machine learning techniques advanced [7]. Videos, unlike static images, provide temporal dynamics and multimodal information, making them richer but also more complex for emotion analysis. In 2005, Hanjalic and Xu [8] proposed a framework to represent and learn the emotional content. Afterward, many researchers began to study the problems in VER. Early studies in the VER field primarily relied on handcrafted features extracted from visual and auditory modalities [9,10]. Because emotions are simultaneously influenced by visual and auditory information, some studies [11,12] focused on fusing audiovisual features to learn complex emotional representations in videos. Recent research in VER has achieved end-to-end learning of emotional representations [13,14] and has explored the utilization of weakly supervised learning techniques [15].

Generally, algorithms for VER can be classified into two distinct groups: (1) handcrafted-feature-based algorithms, which utilize handcrafted emotion features to describe video content; (2) neural-network-based algorithms, which employ neural networks to learn the emotional information of videos from massive data and build emotion models. Despite the rapid progress, VER continues to pose challenges, which are primarily attributed to the subtleties and context-sensitive nature inherent in human emotional expression. The differences in cultural expressions, individual differences in emotional intensity, and the presence of ambiguous or mixed emotions further complicate the recognition process. Furthermore, the temporal dynamics of videos demand that models effectively capture both spatial and temporal dependencies.

This study conducted an in-depth review of recent developments in VER, with a focus on the psychological foundations, datasets, evaluation metrics, and state-of-the-art algorithms. It also identified key challenges and proposed future research directions aimed at addressing these challenges, thereby laying the groundwork for more robust VER systems. The major contributions are summarized below:

- Comparative analysis of classic algorithms: This study systematically evaluated classic algorithms in the VER field, including the comparison and analysis of their performance on four datasets. This evaluation provides a benchmark reference for researchers to select or refine algorithms for specific VER tasks, while highlighting unresolved challenges that require further innovation.
- Comprehensive analysis of challenges: This study comprehensively analyzed the pivotal challenges hindering the development of VER, categorizing them into three interdependent dimensions. Through this analysis, this study identified the root causes of current limitations, established a foundation for future research, and bridged theoretical insights with actionable strategies to advance the field.
- Actionable future research directions: This paper proposes actionable research directions to address the key challenges, thereby promoting the development of the VER field. By systematically linking these directions to the challenges, the paper provides a clear roadmap for future innovations, thereby guiding researchers in designing precise and efficient VER systems.

The subsequent sections are organized as follows: Section 2 and Section 3 present psychological models and benchmark datasets in VER, respectively. Then, Section 4 and Section 5 illustrate methods and results in VER. Moreover, a discussion and conclusion are introduced in Section 6 and Section 7, respectively. Acronyms are reported in Table 1.

## 2. Psychological Models for VER

In the field of psychology, there are two main types of models used to measure emotions, namely, the discrete emotion model and the dimensional emotion model. The discrete emotion model divides emotions into several relatively independent emotion categories, which has the advantages of simplicity. In 1980, Plutchik [48] proposed that humans have eight basic emotion categories, i.e., anger, anticipation, disgust, fear, joy, sadness, surprise, and trust. In 1992, Ekman [49] introduced six basic emotion categories. Moreover, other emotion categories, such as boredom and excitement, have also been used to describe video emotions [50,51]. Recently, more emotion categories have been reported, e.g., 27 categories [52] and 80 categories [53]. Limited by the number of emotion categories, the discrete emotion model has the following drawbacks. First, the discrete emotion model merely represents a restricted range of emotions. Second, there is a certain correlation between some emotion categories, but it is difficult for the discrete emotion model to measure and represent this correlation. Finally, the discrete emotion model cannot describe the process of emotion generation, development, and disappearance.

Some researchers proposed the dimensional emotion models [54,55], where a multidimensional space is utilized to describe continuous emotions. Specifically, the dimensional emotion model employs a dimension in the multidimensional space to describe a certain aspect of emotion and a coordinate point in the space to represent a certain emotional state. Three dimensions are utilized to describe the emotion model, i.e., valence, arousal, and dominance. Among them, the valence dimension measures the degree of happiness of emotions, ranging from depression to ecstasy. The arousal dimension evaluates the intensity of activation and emotional stimulation, ranging from passive to active. The dominance dimension captures the extent of emotional authority and control, and measures the degree of affecting others or being affected by others, ranging from submission to dominance. As the dominance dimension is hard to measure, the two-dimensional emotion model is generally used in the emotion analysis field, which is called the valence–arousal emotion model. Moreover, Russell [54] demonstrated that emotion categories can be mapped to the dimensional emotion model.

## 3. VER Datasets

To advance research in VER, some datasets containing diverse and rich emotional content have been created to train and test emotion recognition methods. These datasets include video clips extracted from movies and social media platforms, ensuring a variety of contexts and cultural backgrounds. The diversity in these datasets is crucial for training robust methods that can generalize across different scenarios. Furthermore, they are annotated with emotional labels or continuous emotion dimensions, providing valuable ground truth for supervised learning tasks. Table 2 reports the datasets’ information for VER, where F1, MSE, and PCC denote the F1 score, mean-square error, and Pearson correlation coefficient, respectively.

DEAP [56] is a multimodal emotion dataset that contains testing videos and audience response data. To collect emotional responses, 32 participants were engaged in viewing 40 music video clips, each lasting one minute. Additionally, facial videos were collected from 22 participants. Due to copyright licensing restrictions, DEAP does not provide the music videos. Links for downloading the videos are public.

MAHNOB-HCI [57] is a multimodal dataset for emotion analysis. This dataset was developed through 27 participants from diverse gender and cultural backgrounds, ensuring extensive emotional responses. MAHNOB-HCI comprises 20 videos, each annotated according to the valence–arousal–dominance model to capture emotional states comprehensively. Moreover, the dataset includes various physiological signals, respiratory amplitude, and skin temperature measurements, providing rich data for analyzing human emotional reactions in depth.

VideoEmotion-8 [9] is structured around the Plutchik emotion model, which categorizes human emotions into eight distinct types. The dataset comprises 1101 videos, which exhibit a wide range of content and contextual variations, guaranteeing robust emotional expression coverage. It provides 10 unique splits of videos, each categorized into training and testing subsets. Roughly two-thirds of the samples within each split are designated for model training, with the residual portions allocated for evaluation purposes. To assess the model performance, VideoEmotion employs the mean accuracy across all 10 testing splits as its primary metric. Overall, VideoEmotion offers a well-structured dataset for investigating emotion recognition in video content.

LIRIS-ACCEDE [29,58] employs the valence–arousal dimensional model to describe emotions, and is a classic dataset for evaluating VER algorithms. From 2015 to 2018, in the movie emotional impact competition organized by MediaEval, the competition organizers used LIRIS-ACCEDE as the competition dataset [60,61,62,63]. Discrete LIRIS-ACCEDE contains 9800 videos from 160 movies. The types of these films are diverse. The cumulative runtime of these clips approximates 73 h, with individual clips varying in length between 8 and 12 s. Each clip is given an emotional label on the valence and arousal dimensions. Continuous LIRIS-ACCEDE [64] selects 30 movies from the 160 movies, which are annotated once per second. In the valence and arousal dimensions, MSE and PCC were utilized as evaluation indicators, respectively.

Ekman-6 [26] is built upon the Ekman model. It includes 1637 videos, which have been carefully annotated to represent the six basic emotions outlined by Ekman’s model. The diversity of sources ensures that the dataset captures a wide range of emotional expressions, cultural contexts, and real-world scenarios. Regarding evaluation, it employs accuracy as a metric for assessing the performance. Ekman-6 constitutes a valuable dataset for promoting VER research.

CAER [16] has an extensive collection of videos, comprising 13,201 clips from 79 TV shows. Each video clip within CAER has been annotated into seven distinct emotion categories. CAER provides a sufficient number of large-scale videos to learn models for context-aware VER. Accuracy is the metric for CAER. In summary, CAER is a valuable dataset for advancing research in VER. Its extensive size and emphasis on context-aware analysis make it an essential tool for developing more sophisticated emotion intelligence technologies.

Video-Danmu [59] contains 4056 clips and 371,177 danmus (i.e., synchronized comments), which are collected from an online video platform. The dataset categorizes each video into one of seven affective categories. Precision and accuracy are utilized as key performance indicators for Video-Danmu. Within the realm of VER, Video-Danmu was the first one to provide the text modality, i.e., synchronized comments.

EEV [20] is the most extensive dataset in VER, including about 1700 h across 23,574 individual videos. The proportions of samples in the training, validation, and test sets are approximately 60%, 15%, and 25%, respectively. In 2021, a challenge was held using EEV as the competition dataset, with PCC as the metric. This vast dataset provides researchers with an unparalleled resource for studying human emotions in depth.

MM-AU [5] contains 8399 video samples, covering a wide range of topics and languages. This dataset offers annotations for 18 topic categories, 3 tone categories, and 2 social message categories, rendering it applicable for three tasks, including VER. These annotations were generated utilizing a semi-automatic approach. F1 and accuracy are the metrics for the MM-AU dataset.

VAD [45] is a comprehensive dataset designed for VER. It sources videos from popular Chinese online platforms, e.g., Bilibili. The dataset contains 19,267 clips, each accompanied by synchronized danmu comments that reflect the viewers’ emotional responses to specific moments in videos. These clips are annotated with emotional labels, enabling research on multi-task learning across different emotion models. The evaluation metrics for VAD are accuracy and F1.

## 4. VER Algorithms

**Figure 1:**
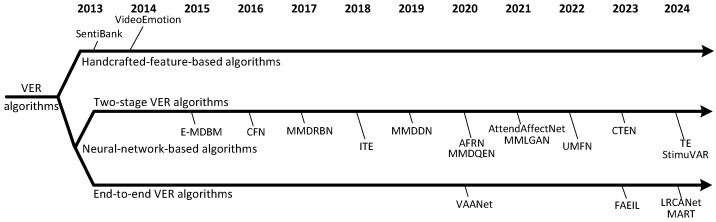
A timeline of classic VER algorithms, namely, SentiBank [65], VideoEmotion [9], E-MDBM [21], CFN [18], MMDRBN [36], ITE [26], MMDDN [34], AFRN [11], MMDQEN [35], AttendAffectNet [66], MMLGAN [37], UMFN [12], CTEN [15], TE [42], StimuVAR [40], VAANet [13], FAEIL [22], LRCANet [4], and MART [14].

Algorithms for VER utilize visual and audio features to infer the emotional reactions of the predominant audiences. Generally, VER algorithms are categorized into two classifications, i.e., handcrafted-feature-based algorithms and neural-network-based algorithms. As shown in Figure 1, before 2015, most VER algorithms predominantly relied on handcrafted features. However, since 2015, neural networks have been progressively adopted in the VER field, and the use of handcrafted features has gradually decreased in this field. Notably, since 2020, end-to-end neural networks have been designed to address the challenges in the VER domain. In recent years, neural networks have become the mainstream and hot technology in this field.

Although the use of handcrafted features is gradually decreasing in this field, they remain effective in scenarios with limited data, constrained computational resources, or high interpretability requirements. With the advent of big data and powerful GPUs, neural networks have demonstrated superior generalization in high-dimensional data (e.g., images, videos) through end-to-end feature learning. Deep neural networks can automatically extract hierarchical features while minimizing manual intervention. Therefore, handcrafted features retain competitiveness in small-sample tasks or lightweight applications, whereas deep learning excels in large-scale and complex tasks.

### 4.1. Handcrafted-Feature-Based Algorithms

**Figure 2:**
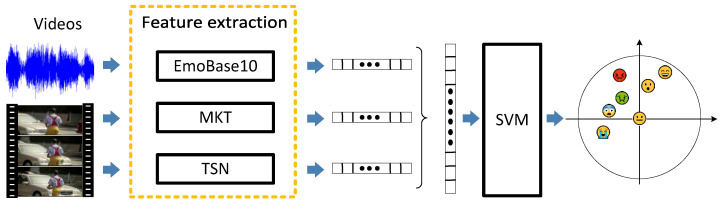
A diagram of MML [10].

In VER, handcrafted-feature-based algorithms utilize handcrafted design features to describe videos, and their core part is video feature extraction and emotion learning. Figure 2 shows a diagram of a classic algorithm [10] based on handcrafted features. Generally, videos contain visual and audio data, from which visual features and audio features can be extracted, respectively. The following will introduce the relevant algorithms in visual emotion features, audio emotion features, and emotion learning.

#### 4.1.1. Visual Emotion Features

Visual features are essential for describing emotions. Based on their capacity to characterize semantic content, visual elements are broadly categorized into LVFs and HVFs. LVFs primarily capture fundamental attributes of video data, such as color, texture, and shape. For VER, commonly used LVFs include GIST [67], LBP [28], HSH [25], DSIFT [19], HOG [24], and self-similarity [68].

HVFs distinguish themselves from LVFs by encoding meaning rather than basic data. While LVFs focus on rudimentary traits, HVFs represent abstract concepts, such as sentiment information, scene information, object information, and motion information. For VER, HVFs mainly encompass Classemes [69], ObjectBank [70], SentiBank [65], MKT [71], and TSN [44].

Generally, different visual features possess distinct capabilities for describing emotions. To determine which visual features can describe video emotions more effectively, comparative experiments were performed on several datasets. On VideoEmotion-8, Jiang et al. [9] compared four LVFs, i.e., GIST, LBP, HOG, and DSIFT. Among them, they discovered that DSIFT obtained relatively better experimental outcomes. On LIRIS-ACCEDE, Baecchi et al. [72] evaluated five HVFs, namely, VGG-FC7 [46], VGG-FC8 [46], Sent-FC7 [73], Sent-FC8 [73], and deep face [74], and found that Sent-FC8 better characterized the emotions in videos. Moreover, Guo et al. [75] also assessed two features based on the VGG [46] network, i.e., OVGG and OFVGG [75], and discovered that OFVGG achieved relatively better experimental results. Furthermore, Yi et al. [10] appraised four visual features, namely, DSIFT, HSH, MKT, and TSN, and found that TSN and MKT performed better in the two dimensions separately.

According to the above findings, several key conclusions were drawn. First, HVFs generally demonstrate a superior ability to describe emotions compared with LVFs. Second, the integration of diverse feature types tends to enhance performance. While expanding the quantity of features alone may not guarantee enhanced performance, the integration of complementary features (e.g., combining low-level texture details with high-level object semantics) promotes predictive accuracy. By leveraging diverse but complementary information, fusion mitigates individual feature limitations, captures multidimensional correlations, and reduces the influence of any single feature.

#### 4.1.2. Audio Emotion Features

The sound in videos can affect the emotions of audiences. In VER, audio features are also crucial. Audio data contains both semantic information and non-semantic information, each of which contributes to emotional perception. The semantic information of audio generally refers to the semantic content conveyed by the sound, while the non-semantic information of audio mainly includes the tone, melody, rhythm, timbre, and speech rate.

Generally, audio features are classified into two categories, namely, LAFs and HAFs. In previous VER studies, LAFs mainly included MFCC [31] and ZCR [47]. HAFs are generally based on LAFs and describe audio data at a higher level. For VER, HAFs mainly include EmoLarge [76], EmoBase10 [77], IS13 [78], GeMAPS [23], SoundNet [79], and VGGish [80].

Different audio features have varying abilities to describe emotions. To find out which audio features are more suitable for emotional description, LIRIS-ACCEDE was employed by the paper [10] to evaluate four audio features, namely, MFCC, EmoLarge, EmoBase10, and IS13. Through the comparative experiments, EmoBase10 attained relatively superior performance. Moreover, Guo et al. [75] compared seven audio features, namely, MFCC, IS13, EmoBase10, EmoLarge, GeMAPS, SoundNet, and VGGish, and discovered that SoundNet and VGGish obtained superior performance in the arousal and valence dimensions, respectively.

The following inferences can be drawn. First, HAFs generally tend to yield better performance than LAFs. This is because HAFs are generally constructed based on LAFs, which improves their generalization capability. Second, exploring the extraction of HAFs from large datasets is a valuable research direction.

#### 4.1.3. Emotion Learning

Handcrafted-feature-based algorithms can model emotional content through classic machine learning algorithms. The learning algorithms employed in previous relevant studies mainly include HMM, SVM [41], RBM [38], MLP [33], LAR [27], and passive aggressive [81].

According to the valence–arousal model, Xu et al. [82] designed a hierarchical method and learned emotional information by utilizing HMM. Baecchi et al. [72] adopted emotion-related features to depict videos and used SVM to learn emotional content. On LIRIS-ACCEDE, Yi et al. [10] evaluated four learning algorithms and discovered that the method using SVM obtained relatively better performance under the same experimental conditions. In the arousal and valence dimensions, Guo et al. [75] utilized the passive-aggressive and least-angle regression algorithms to predict emotion, respectively. Because SVM possesses strong generalization capabilities, many VER algorithms utilize SVM to learn emotion models.

In summary, approaches utilizing handcrafted features and traditional machine learning algorithms have achieved some success in the domain of VER. According to visual theory, researchers designed features and customized them for the characteristics of video data. While handcrafted features offer high customizability, these features rely on shallow linear structures and have difficulty in effectively modeling large-scale and complex data. On large-scale datasets, such algorithms often attain less-than-satisfactory performance. Therefore, neural networks have been increasingly adopted in this field.

### 4.2. Neural-Network-Based Algorithms

In VER, neural-network-based algorithms describe video content through neural networks, with the core being the design and training of neural networks. Depending on whether there exists an independent feature extraction module, these algorithms are typically classified into two major categories, i.e., two-stage VER algorithms and end-to-end VER algorithms.

#### 4.2.1. Two-Stage VER Algorithms

According to the order of video data being modeled, the two-stage VER algorithms primarily include two steps. Initially, feature vectors are derived from video data. Subsequently, a neural network architecture is constructed, and the model undergoes training utilizing these vectors as input. Some classic algorithms are introduced below.

Generally, there exists a nonlinear relationship between different modes. E-MDBM [21] was designed to model nonlinear relationships between modalities. E-MDBM contains three separate paths (i.e., vision, auditory, and text), with each composed of stacked restricted Boltzmann machines to learn modality-specific representations. By combining these paths, the model captures the nonlinear relationship between modalities. The final representation is a shared embedding space where statistical properties from different modalities are unified. In this representation, high-level semantic correlations between the three paths are leveraged to represent videos.

To effectively fuse event, object, and scene features, Chen et al. [18] designed CFN. Pre-trained models from external datasets are leveraged to extract vectors of the three features. Following feature extraction, these vectors are processed by using L1 and RootSift [83] normalizations. These normalized features are then integrated through CFN to produce accurate predictions. CFN mainly contains linear and softmax layers.

To mine the correlation between visual and audio modalities, MMDRBN [36] was designed. By minimizing the KL divergence, MMDRBN is transformed into a multimodal inference deep network. During training, the back propagation strategy is utilized to optimize MMDRBN. During testing, the trained model is employed to predict emotional labels of videos. To address the limitations of MMDRBN, Wang et al. [84] proposed an improved version called knowledge-augmented MMDRBN. By constructing a representation that bridges visual and auditory elements with semantic attributes, the model achieves a more comprehensive understanding of emotional expression in videos.

The labels of VER datasets have inherent noise, which poses a significant challenge for obtaining reliable supervised data to train emotion models effectively. MMDDN [34] was devised to solve this problem. By leveraging the embedding network, MMDDN fuses rich multimodal information. LSTM [30] is used to predict the label of a sample. Moreover, MMDQEN [35] further improved MMDDN. MMDQEN adopts a non-parametric approach instead of relying on LSTM, which significantly reduces the computational burden associated with fusing temporal information. By inferring potential labels from noisy training samples, MMDQEN provides more accurate annotations for emotional classifiers.

**Figure 3:**
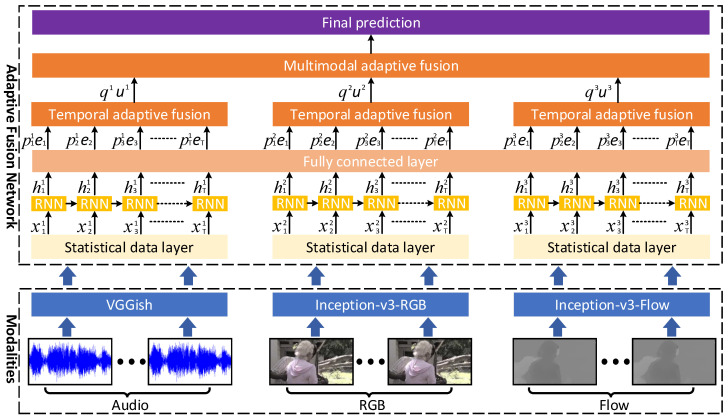
A diagram of AFRN [11].

Humans perceive the world through multimodal information, and their emotional states are influenced by previously encountered visual scenes and auditory cues. Therefore, understanding the relative importance of multiple modalities and the temporal relationships within inputs is crucial for accurately predicting emotions in videos. To leverage these relationships effectively, AFRN was proposed by [11]. As illustrated in Figure 3, three layers were designed for extracting robust input features, integrating temporal information, and combining information across different modalities.

To address the challenge of multimodal fusion in VER, MMLGAN [37] was introduced. This approach extends traditional attention mechanisms to facilitate multi-level data integration and enhances the representation of videos by designing a multimodal fusion unit. This unit operates in two stages: local and global attention. The local attention stage selects key components from various streams, ensuring that the most relevant features are highlighted. During the global attention stage, it captures the temporal distribution of information, providing a comprehensive understanding of the video content over time. MMLGAN selectively emphasizes crucial emotional elements, thereby improving the overall representation of emotions in videos.

By leveraging self-attention to capture relationships across modalities and time, AttendAffectNet [66] was designed to systematically analyze viewers’ emotional reactions. Specifically, three variants were designed. The feature AttendAffectNet, which utilizes self-attention on multimodal features to capture inter-modal relationships, achieves superior performance between the three variants. Through the innovative usage of self-attention mechanisms, AttendAffectNet learns the complex interactions between different modalities, thereby enhancing the accuracy of emotional response prediction.

To address the issue of ignoring contextual cues in videos, CAF [17] was proposed. Key regions are extracted to capture the emotional cues contained in videos. RPN is utilized to derive features from key regions, facilitating the construction of an emotional similarity graph. To enhance the effectiveness, FNN is used to allocate weights to diverse regions based on their emotional significance, followed by a GCN, which elucidates the interconnections between these key regions. Additionally, MFCC is extracted from the auditory modality to complement the visual modality.

In the EEV challenge held in 2021, methods [85,86,87] won first, second, and third places, respectively. Among them, Huynh et al. [85] used EfficientNet [88] and TRILL [43] to calculate visual and auditory features. The temporal convolutional network was designed to learn temporal relationships. Lin et al. [86] utilized Swin-L [89] and VGGish to extract visual and auditory features. Meanwhile, two layers of bidirectional GRUs are proposed to build the emotion model. Yan et al. [87] used Inception-Resnet-v2 [90] and S3D [39] to calculate visual characteristics, employed VGGish to extract auditory features, and proposed a fusion block to combine the above characteristics.

By integrating visual–audio representations, deep graph fusion [91] was proposed for estimating the evoked expressions of viewers. First, salient feature vectors are extracted from videos via pre-trained models. Second, the vectors are fed to the graph structure and processed through a GCN to generate node embeddings. Third, integration mechanisms are applied to synthesize the depictions from the audiovisual pathways. Ultimately, the resultant embeddings are leveraged to estimate scores of samples. Moreover, a semantic embedding loss is incorporated to enhance the performance.

**Figure 4:**
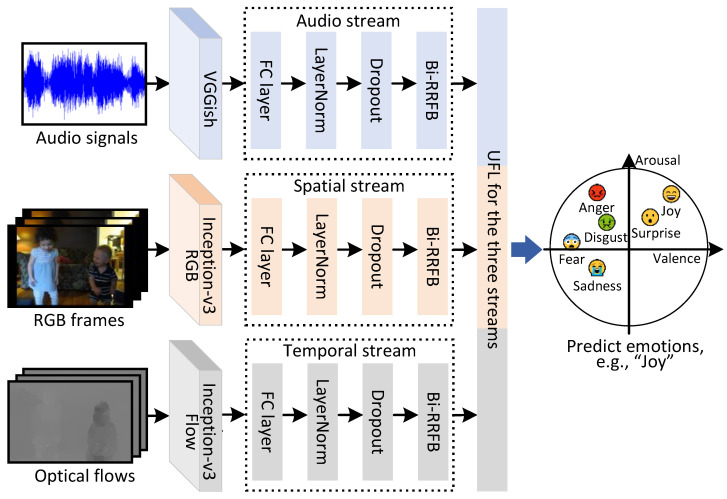
A diagram of UMFN [12].

By fusing multimodal features from multiple stages, UMFN [12] was devised to improve AFRN. As shown in Figure 4, three modalities of video are utilized as the input into UMFN, and the unified fusion layer was designed to fuse the output information. Multiple modalities are integrated by UMFN, thereby enhancing the model’s capability to characterize video-based emotional content.

Based on weakly supervised learning, CTEN [15] was proposed to equip the network with contextual awareness for interpreting emotional expressions. The 3D Resnet-101 and 2D Resnet-50 are utilized to calculate characteristics for audiovisual streams. Then, a module is introduced to learn the temporal relationship of two modalities. By choosing keyframes, the model concentrates on contexts containing complementary information.

To overcome the limitations of MLLMs, which predominantly focus on semantic video content, an approach named StimuVAR [40] has been introduced. This approach includes two-level awareness, i.e., frame-level awareness, which involves sampling video frames likely to elicit emotional responses, and token-level awareness, which executes tube selection to focus on emotion-triggered spatiotemporal regions. Additionally, instruction data has been created to conduct affective training, guiding MLLMs’ reasoning capabilities toward emotional focus.

To reduce the noise in the temporal domain, TE [42] is introduced. This method takes motion, semantic, and audio modalities as the input. A module is designed to enhance the temporal information of the modalities. Through the interaction with these modalities, this module enhances the key temporal information and suppresses the irrelevant information.

In summary, the above algorithms use the two-stage framework to build a video emotion model. The primary research directions in this domain concentrate on three main aspects: first, exploring or identifying features that are more suitable for describing emotions in videos; second, researching more efficient temporal modeling algorithms; and third, investigating improved methods for effectively fusing multiple features.

#### 4.2.2. End-to-End VER Algorithms

Unlike two-stage VER algorithms, end-to-end VER algorithms implement the extraction of video features and the construction of emotion models in a network and ultimately achieve the prediction of video labels. Due to limitations such as GPU memory and sample data size, it is challenging to jointly train long-range visual and audio models in a single network.

VAANet [13] was designed to implement an end-to-end scheme for VER. In VAANet, three attention strategies are integrated into the visual 3D CNN. Concurrently, temporal attentions are added to the audio 2D CNN. Under the polarity–emotion hierarchy constraint, a loss function is formulated to steer the attention mechanism. This network constitutes the first end-to-end model within the domain of VER.

A network [22] was designed to learn frame-level emotional information. Regarding the architecture, ResNet-50 [92] was selected as the backbone. Frame-level features are fused via the pooling method to create video-level representations. Through the alignment of cross-domain features, the network can obtain knowledge from the source dataset while maintaining adaptability to the target video frames.

**Figure 5:**
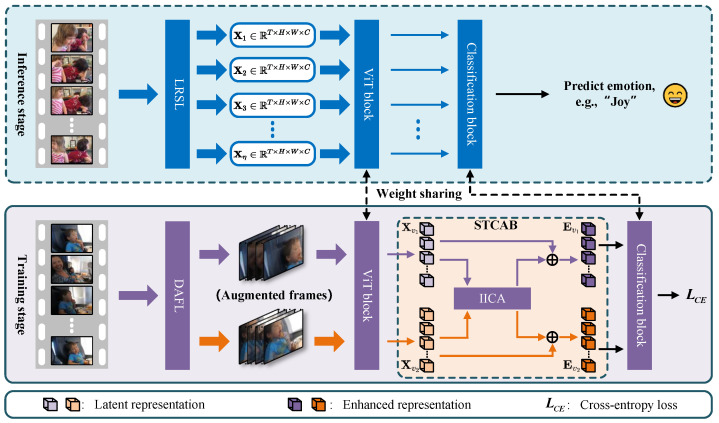
A diagram of LRCANet [4].

To address the challenge of encoding long-range contextual correlations in videos, LRCANet [4] was proposed. A diagram of LRCANet is visualized in Figure 5. A spatio-temporal correlation-aware block was devised to capture long-range relations between input tokens, where local correlations are learned through convolutional layers, and spatio-temporal relationships are learned by the inter-image cross-attention. To enhance sample diversity, a dual-augmentation fusion layer is introduced, which integrates each frame with its corresponding temporal counterpart. Furthermore, a long-range sampling layer is devised to create samples spanning extensive spatial and temporal domains, ensuring rich and varied representations.

To address the long-standing issue of limited training data for VER, a masked learning framework named MART [14] was proposed. The core idea is to learn robust VER representations based on the MAE paradigm. First, emotional cues are extracted, and the reliability of emotional cues is verified by calculating the matching degree between emotional dictionaries and videos. Second, a masked strategy is proposed to reconstruct the temporal distribution of the masked segments. Finally, cross-modal attention is utilized to construct a complementary learning block. Without requiring the incorporation of additional large-scale datasets, MART learns the emotional cues from video content.

Several challenges in the field of VER have been addressed through various methods, which have yielded significant advancements and established foundational approaches. However, certain limitations and unresolved issues still persist within this domain, requiring further exploration and refinement. This work undertakes an examination of these existing problems, with a particular focus on identifying potential research directions for future investigations.

## 5. Results

In the field of VER, the datasets frequently employed to assess algorithm performance are the MediaEval 2015 task of LIRIS-ACCEDE, EEV, VideoEmotion-8, and Ekman-6. On these four datasets, this work compared the experimental results of a series of classic methods and drew relevant inferences. Specifically, Table 3 shows the results of classic methods on the MediaEval 2015 task of LIRIS-ACCEDE, where ACC denotes accuracy. Table 4 compares the PCC values of related algorithms on EEV, Table 5 reports the accuracy values of relevant methods on VideoEmotion-8, and Table 6 summarizes the accuracy values of classic methods on Ekman-6. Moreover, the experimental results presented in these tables were sourced from the corresponding papers of these methods.

As summarized in Table 3, experimental outcomes indicate diverse performance across different approaches for VER. The majority of methods leverage both visual and audio modalities, with a few relying solely on visual features. In terms of the arousal dimension, the accuracy ranged from 45.04% to 60.88%, while the valence prediction presented a range of 36.12% to 48.61%. Notably, methods such as MLG-S and AFRN achieved superior performances, where AFRN reached 58.22% for arousal and 48.61% for valence. This indicates that multimodal approaches, particularly those integrating advanced deep learning architectures, tended to outperform the unimodal methods. The results also demonstrate a gradual improvement in accuracy over time, likely due to advancements in model design and feature extraction techniques. However, compared with arousal, the performance of valence prediction was relatively lower, which might suggest inherent challenges or dataset biases in this dimension. Overall, these findings highlight the effectiveness of multimodal fusion strategies and the potential for further improvements in VER models.

Table 4 presents the results of classic methods on EEV, with the results measured using PCC. All methods utilize both visual and audio modalities. The PCC values ranged from 0.00819 to 0.05477 for TCN, indicating that while all the methods showed some ability to capture emotional correlations, their effectiveness is limited. Specifically, TCN achieved the highest PCC scores, indicating that the architecture of TCN better models the temporal or multimodal information of emotions on this dataset. Despite employing both visual and audio modalities, these methods exhibited modest performances on EEV, highlighting the complexity of VER in this context.

Table 5 reports the results of classic methods on the VideoEmotion-8 dataset, with accuracy values that ranged from 40.50% to 59.39%. Notably, TE [42] achieved the highest accuracy at 59.39%, closely followed by FAEIL [22], LRCANet [4], and CTEN [15] at 57.63%, 57.40%, and 57.30%, respectively. Most methods concurrently utilized audiovisual modalities, underscoring the critical role of cross-modal fusion within the VER framework. Methods relying only on the visual modality, e.g., KeyFrame [106] and DFAN [107], generally performed less effectively, where they achieved performances of 52.85% and 53.34%, respectively. This underscores the benefit of combining multiple modalities to enhance the recognition accuracy. In conclusion, technologies focusing on multimodal fusion, temporal modeling, and advanced network architectures play a crucial role in achieving outstanding results.

As reported in Table 6, the performance reveals significant variations across different approaches, where FAEIL [22] achieved the highest accuracy at 60.44%, followed closely by LRCANet [4] and KeyFrame [106] at 59.78% and 59.51%, respectively. Notably, methods such as CFN [18], DFAN [107], and KeyFrame [106], which rely solely on the visual modality, still achieved relatively high performances, suggesting that visual features alone can be highly effective on this dataset. Overall, these discoveries accentuate the significance of model design and feature extraction strategies, along with the potential advantages of concentrating on visual information in some circumstances.

The aforementioned methods mainly adopted three types of network architectures, i.e., LSTM, a CNN, and Transformer. In the field of VER, the comparison of the three types of architectures is presented below:

- LSTMs leverage a recurrent structure to process data sequentially, capturing long-term temporal dependencies. In the VER field, LSTMs learn contextual information from previous frames to infer emotions. However, long videos may still suffer from partial forgetting. Moreover, LSTMs are limited in spatial modeling, and their sequential processing leads to slow training.
- CNNs use kernels to extract local features and build hierarchical abstractions via stacked layers, which enables them to learn video content efficiently. Their limitations include limited receptive fields and requiring deep stacking for long-range dependencies. In practice, the kernel size and depth must be carefully designed, and 3D convolutions are essential for video data.
- Transformers employ self-attention to learn dependencies across elements in a video, thereby establishing cross-temporal relationships. Their primary strength lies in building long-range dependency models. However, their quadratic complexity in attention matrices limits long-sequence processing. In practice, local or sparse attention variants can reduce costs, and positional encodings are critical for videos.

Attention mechanisms have achieved significant progress in affective computing, and various related techniques have been customized for specific task requirements. For example, MMLGAN [37] captures the correlations between multiple modalities through a fusion block and an improved attention mechanism. AttendAffectNet [66] explores feature-level and temporal-level interactions of multimodal inputs via self-attention. Moreover, the hierarchical attention [108] is devised to improve discriminative features, thereby enabling the effective fusion of multiple features. This diversification of these techniques highlights the flexibility of attention mechanisms and emphasizes the necessity of domain-specific optimization in the context of affective computing.

From these tables, the following conclusions can be inferred. First, methods that utilize visual and audio modalities tend to achieve higher performance compared with those relying solely on a single modality. Second, the design of the model architecture plays a vital role in determining the performance. Advanced architectures, such as those integrating temporal modeling and multimodal fusion techniques, surpass simpler models. Third, although many studies have made a series of advances in this field, the accuracy of VER remains relatively low.

## 6. Discussions

### 6.1. Challenges

The domain of VER has witnessed notable advancements over the past decade. However, there are still several key scientific challenges that require further exploration and research, as follows.

#### 6.1.1. Gaps Between Emotional Representations and Labels

In terms of theoretical research, the direct connection between video emotional representations and labels remains unclear, which makes it difficult to attain better experimental results on benchmark datasets. Human emotions are often complex and context-dependent. VER requires capturing subtle emotional cues from both visual and auditory modalities, but existing emotional representations may overlook complex emotional cues.

Moreover, current models may not be able to fully capture context. Similar visual or auditory cues can evoke different emotions in different contexts, leading to prediction errors when the context is ignored. Therefore, one of the critical challenges in VER is the emotional gaps between representations and labels. How to design an excellent emotional network model to establish a direct connection between representations and labels is a problem worthy of further research.

#### 6.1.2. Large-Scale and High-Quality VER Datasets

Compared with objective tasks, such as image classification, building large-scale, high-quality VER datasets poses significant challenges. The subjectivity of emotions introduces great complexity in data annotation, as different annotators may have different perceptions of the same video due to psychological or cultural differences. This inherent subjectivity can lead to potential label inconsistencies, which can reduce the generalization ability if left unaddressed.

The main research significance lies in developing innovative methods to effectively annotate large-scale VER datasets while minimizing label noise. Given the limitations of human and financial resources, traditional manual annotation methods are impractical for constructing large-scale datasets. Therefore, exploring intelligent algorithms to assist the annotation process becomes crucial. Such algorithms may include active learning strategies and weakly supervised strategies.

Furthermore, addressing the label noise problem is essential for maintaining data quality. Techniques such as multi-annotator consensus mechanisms or statistical methods for identifying and correcting noisy labels can significantly improve the reliability of VER datasets. These advancements not only benefit VER but also extend to other domains involving subjective evaluations, making this research direction highly valuable in the broader application of machine learning and affective computing. In conclusion, developing efficient annotation methods and noise reduction strategies for large-scale VER datasets is crucial for advancing VER technologies.

#### 6.1.3. Efficient Integration of Multiple Modalities

Videos typically contain both visual and auditory modalities, which jointly influence the emotions of viewers. Current research has not fully explored the intricate correlations between multiple modalities, particularly in three aspects, namely, temporal alignment, dynamic modality weighting, and noise control.

First, temporal alignment between modalities presents significant complexities. For instance, video data is typically sampled at 30 FPS, while audio is captured at 16 kHz, leading to discrepancies in time-sensitive emotional cues. This mismatch can degrade the recognition accuracy. To address this, dynamic time warping mechanisms can be employed to align multimodal temporal sequences and ensure consistency at critical time points, mitigating errors caused by sampling rate differences.

Second, the contribution of modalities to emotional expression varies. For example, visual modality is more informative for detecting anger or happiness, while audio modality is critical for identifying sadness or anxiety. Assigning equal weights to all modalities may lead to suboptimal performance by overlooking dominant features. Therefore, dynamic weighting strategies, such as attention mechanisms or uncertainty modeling, may be essential to adaptively adjust the modality importance.

Third, multimodal fusion often introduces redundant or conflicting information. For example, background noise in videos may confuse visual feature extraction, irrelevant text could mislead emotional judgments, and imbalances in data volume across modalities may cause overfitting to noise. To alleviate these issues, techniques like sparse representations can filter redundant features, and cross-modal filtering mechanisms can eliminate contradictions, thus enhancing the model’s robustness.

Through the above strategies, multiple modalities can be efficiently integrated, thereby building a more accurate VER system. The significance of the research lies in developing an efficient multimodal fusion architecture to handle the aforementioned issues. These advancements will not only improve the performance of VER models but also contribute to a deeper understanding of multimedia content. By addressing this challenge, researchers can create more powerful VER systems capable of capturing subtle emotional cues in real-life situations. This will significantly promote progress in fields such as emotion-centric technologies and personalized content recommendation.

### 6.2. Future Work

Future orientations in VER focus on addressing the key challenges, including the gaps between emotional representations and labels, the efficient integration of multiple modalities, and the establishment of high-quality datasets. These challenges are critical to advancing VER technologies and improving their real-world applications.

First, bridging the gap between emotional representations and labels requires innovative approaches to better capture the nature of human emotions. Future research could explore advanced neural network architectures, such as graph-based models or hierarchical attention [108], to model the subtle relationships between visual and auditory cues and their corresponding labels. Furthermore, integrating emotional psychological theories into the design of representation learning frameworks could help create more interpretable and effective VER systems.

Second, building large-scale and high-quality VER datasets is essential for training robust models. To address the subjectivity of emotions and reduce label noise, researchers should explore active learning strategies to selectively annotate samples with high uncertainty and utilize weakly supervised techniques to generate reliable labels on a large scale. Additionally, exploring automated annotation methods, such as using pre-trained models or crowdsourcing frameworks with quality control mechanisms, could significantly alleviate the burden of manual annotation. Methods for identifying and mitigating noisy labels, such as multi-annotator consensus approaches or statistical noise reduction approaches, should also be essential to ensure the reliability of datasets.

Third, enhancing the integration of multiple modalities is crucial for promoting the performance of VER systems. Future research should focus on designing multimodal fusion architectures that can effectively align and integrate visual and auditory modalities in both spatial and temporal domains. For example, cross-modal Transformers or attention-based mechanisms could be developed to capture the complementarity of multimodalities and model their temporal relationships more accurately. Moreover, exploring modality-specific processing techniques, such as separate encoders for visual and auditory inputs followed by adaptive fusion layers, could help maximize the contribution of each modality to VER.

In conclusion, addressing these challenges will not only advance VER technologies but also have a broader influence on emotion-centric applications and multimedia analysis. By exploring more sophisticated models, datasets, and multimodal integration strategies, researchers can create VER systems that are more accurate, reliable, and capable of capturing emotions expressed in videos.

## 7. Conclusions

VER is a crucial research domain in affective computing, possessing considerable social significance and vast application potential. Many scholars have attained research outcomes in the aspects of emotional psychological models, VER datasets, and VER algorithms. Regarding the aforementioned three aspects, this paper elaborately expounds on the relevant research advancements in the field of VER, filling the gap in the survey of this domain.

On the basis of investigations and analysis, this paper puts forward the current challenges confronted in this field, including gaps between emotional representations and labels, large-scale and high-quality VER datasets, and the efficient integration of multiple modalities. Moreover, this paper predicts the potential research directions, such as advanced neural network architectures, efficient multimodal fusion strategies, high-quality emotional representation, and robust active learning strategies.

## Figures and Tables

**Table 1:** Acronyms and descriptions.

Acronym	Description
AFRN	Adaptive fusion recurrent network [11]
CAER	Context-aware emotion recognition [16]
CAF	Context-aware framework [17]
CFN	Context fusion network [18]
CNN	Convolutional Neural Network
CTEN	Cross-modal temporal erasing network [15]
DSIFT	Dense scale invariant feature transform [19]
EEV	Evoked Expression in Video [20]
E-MDBM	Enhanced multimodal deep Boltzmann machine [21]
FAEIL	Frame-level adaptation and emotion intensity learning [22]
FNN	Feedforward neural network
GCN	Graph convolutional network
GeMAPS	Geneva minimalistic acoustic parameter set [23]
GRUs	Gated recurrent units
HAFs	High-level audio features
HMM	Hidden Markov model
HOG	Histogram of oriented gradient [24]
HSH	Hue–saturation histogram [25]
HVFs	High-level visual features
ITE	Image Transfer Encoding [26]
LAFs	Low-level audio features
LAR	Least-angle regression [27]
LBP	Local binary pattern [28]
LIRIS-ACCEDE	LIRIS annotated creative commons emotional database [29]
LRCANet	Long-range correlation-aware network [4]
LSTM	Long short-term memory [30]
LVFs	Low-level visual features
MART	Masked affective representation learning [14]
MFCC	Mel-frequency cepstrum coefficients [31]
MKT	Motion keypoint trajectory
MLG-S	Multimodal local–global system [32]
MLLMs	Multimodal large language models
MLP	Multi-layer perceptron [33]
MM-AU	Multimodal ads understanding [5]
MMDDN	Multimodal deep denoise network [34]
MMDQEN	Multimodal deep quality embedding network [35]
MMDRBN	Multimodal deep regression Bayesian network [36]
MML	Multimodal learning [10]
MMLGAN	Multimodal local–global attention network [37]
MSE	Mean-square error
PCC	Pearson correlation coefficient
RBM	Restricted Boltzmann machine [38]
RPN	Region proposal network
S3D	Separable 3D CNN [39]
StimuVAR	Stimuli-aware video affective reasoning [40]
SVM	Support vector machine [41]
TE	Temporal enhancement [42]
TRILL	Triplet loss network [43]
TSN	Temporal segment network [44]
UMFN	Unified multi-stage fusion network [12]
VAANet	Visual audio attention network [13]
VAD	Video affective dataset [45]
VER	Video emotion recognition
VGG	Visual geometry group [46]
ZCR	Zero-crossing rate [47]

**Table 2:** Datasets for VER.

Dataset	Year	Emotion Model	Main Evaluation Metrics
DEAP [56]	2012	Valence–arousal–dominance	Accuracy
MAHNOB-HCI [57]	2012	Valence–arousal–dominance	F1
VideoEmotion-8 [9]	2014	Plutchik	Accuracy
LIRIS-ACCEDE [29,58]	2015	Valence–arousal	MSE, PCC, and accuracy
Ekman-6 [26]	2018	Ekman	Accuracy
CAER [16]	2019	6 emotions	Accuracy
Video-Danmu [59]	2020	7 emotions	Precision and accuracy
EEV [20]	2021	5 emotions	PCC
MM-AU [5]	2023	3 emotions	F1 and accuracy
VAD [45]	2024	Valence–arousal and 13 emotions	F1 and accuracy

**Table 3:** Results of classic methods on the MediaEval 2015 task of LIRIS-ACCEDE.

Method	Visual	Audio	Arousal (%)	Valence (%)
Mironica et al. [93]	✓	✓	45.04	36.12
Thomas et al. [94]	✓	✓	48.20	44.64
Dai et al. [95]	✓	✓	48.84	41.78
Chakraborty et al. [96]	✓	✓	48.95	35.66
Marin et al. [97]	✓		51.89	38.54
Seddati et al. [98]	✓	✓	52.44	37.28
Trigeorgis et al. [99]	✓	✓	55.72	41.48
Lam et al. [100]	✓	✓	55.91	42.96
Baecchi et al. [72]	✓		55.98	45.31
MMDDN + MMCLF [34]	✓	✓	56.75	45.03
MMDQEN [35]	✓	✓	56.75	45.03
OFVGG + GeMAPS [101]	✓	✓	57.00	40.83
MML [10]	✓	✓	57.40	46.22
Zhang et al. [102]	✓	✓	57.50	45.90
MLG-S [32]	✓	✓	57.90	48.20
AFRN [11]	✓	✓	58.22	48.61
Wang et al. [103]	✓	✓	60.88	43.74

**Table 4:** Results of classic methods on EEV.

Method	Visual	Audio	PCC
Ho et al. [91]	✓	✓	0.00819
MGN-MA [87]	✓	✓	0.02292
Lin et al. [86]	✓	✓	0.04430
TPF [104]	✓	✓	0.05400
TCN [85]	✓	✓	0.05477

**Table 5:** Results of classic methods on VideoEmotion-8.

Method	Visual	Audio	ACC(%)
StimuVAR [40]	✓		40.50
Peng et al. [105]	✓	✓	43.20
MART [14]	✓	✓	50.83
MMLGAN [37]	✓	✓	51.14
ITE [26]	✓	✓	52.60
CAF [17]	✓	✓	52.70
KeyFrame [106]	✓		52.85
DFAN [107]	✓		53.34
VAANet [13]	✓	✓	54.50
UMFN [12]	✓	✓	55.80
CTEN [15]	✓	✓	57.30
LRCANet [4]	✓	✓	57.40
FAEIL [22]	✓	✓	57.63
TE [42]	✓	✓	59.39

**Table 6:** Results of classic methods on Ekman-6.

Method	Visual	Audio	ACC(%)
ITE [26]	✓	✓	51.20
CFN [18]	✓		51.80
MART [14]	✓	✓	52.66
CAF [17]	✓	✓	53.60
VAANet [13]	✓	✓	55.30
DFAN [107]	✓		57.37
CTEN [15]	✓	✓	58.20
KeyFrame [106]	✓		59.51
LRCANet [4]	✓	✓	59.78
FAEIL [22]	✓	✓	60.44
